# Deregulation of Adaptive T Cell Immunity in Multiple Myeloma: Insights Into Mechanisms and Therapeutic Opportunities

**DOI:** 10.3389/fonc.2020.00636

**Published:** 2020-05-05

**Authors:** Noémie Leblay, Ranjan Maity, Fajer Hasan, Paola Neri

**Affiliations:** Arnie Charbonneau Cancer Institute, University of Calgary, Calgary, AB, Canada

**Keywords:** multiple myeloma, immunotherapy, bone marrow microenviroment, monoclonal antibodies, T-cell therapies

## Abstract

Immunotherapy has recently emerged as a promising treatment option for multiple myeloma (MM) patients. Profound immune dysfunction and evasion of immune surveillance are known to characterize MM evolution and disease progression. Along with genomic changes observed in malignant plasma cells, the bone marrow (BM) milieu creates a protective environment sustained by the complex interaction of BM stromal cells (BMSCs) and malignant cells that using bidirectional connections and cytokines released stimulate disease progression, drug resistance and enable immune escape. Local immune suppression and T-cell exhaustion are important mediating factors of clinical outcomes and responses to immune-based approaches. Thus, further characterization of the defects present in the immune system of MM patients is essential to develop novel therapies and to repurpose the existing ones. This review seeks to provide insights into the mechanisms that promote tumor escape, cause inadequate T-cell stimulation and impaired cytotoxicity in MM. Furthermore, it highlights current immunotherapies being used to restore adaptive T-cell immune responses in MM and describes strategies created to escape these multiple immune evasion mechanisms.

## Introduction

Multiple myeloma (MM), although a rare disease, is the second most common hematologic malignancy (1) with over 130,000 new cases occurring every year globally (2). It is a cancer of plasma cells, resulting from abnormal growth of malignant plasma cells in the bone marrow (BM) (3). MM is associated with impaired immunity and immune dysregulation. As such the B-cell dysfunction is characterized by immunoparesis, hypo-gammaglobulinemia of the uninvolved immunoglobulins and increased susceptibility to infections (4). Deficiencies in T-cell function and tissue distribution have been also reported in MM (5). A well-recognized feature of MM is also the bidirectional relationship between the tumor plasma cells and the BM milieu, which provides a protective niche promoting MM tumor growth and loss of immune surveillance (6). Although the advent of novel therapies has improved the outcomes of MM patients (7, 8), the majority of them will relapse and became refractory to current therapy. Therefore, innovative therapeutic strategies, such as immunotherapy, have been established to improve the survival of these patients (9, 10). In the last ten years, a deeper insight into MM biology and its immune defects alongside with the development of numerous immune-based therapies have allowed immunotherapy to become a promising new treatment option for MM patients (9, 10).

As such three major anti-MM immunotherapeutic approaches have been developed: (i) agents that remove the breaks of the immune system, such as immunomodulatory agents (IMiDs) and immune checkpoint inhibitors, (ii) agents that target highly selective antigens on the MM cells in the form of monoclonal antibodies (mAbs) and (iii) agents that stimulate immune cells to selectively kill the malignant cells, such as chimeric antigen receptor (CAR) T-cells, bispecific T-cell engagers (BiTE), and anti-MM vaccines. Those strategies have shown encouraging results in patients with relapsed refractory MM (RRMM) and hold the potential of targeting specifically the malignant cells and the stimulation of a continued response due to harnessing immune surveillance against MM. Nevertheless, the field still presents many challenges, such as the need for tailored therapeutic strategies and biomarkers, the difficulty of selecting the appropriate combination therapy, and resistance to currently available immune-based approaches. Here, we will review the mechanisms that lead to immunosuppression and reduce immune recognition in MM and highlight the strategies created to escape these multiple immune evasion mechanisms to provide long term disease control and better survival for MM patients.

## Immune Dysfunction in MM

### Local Immune Suppression

**Figure 1:**
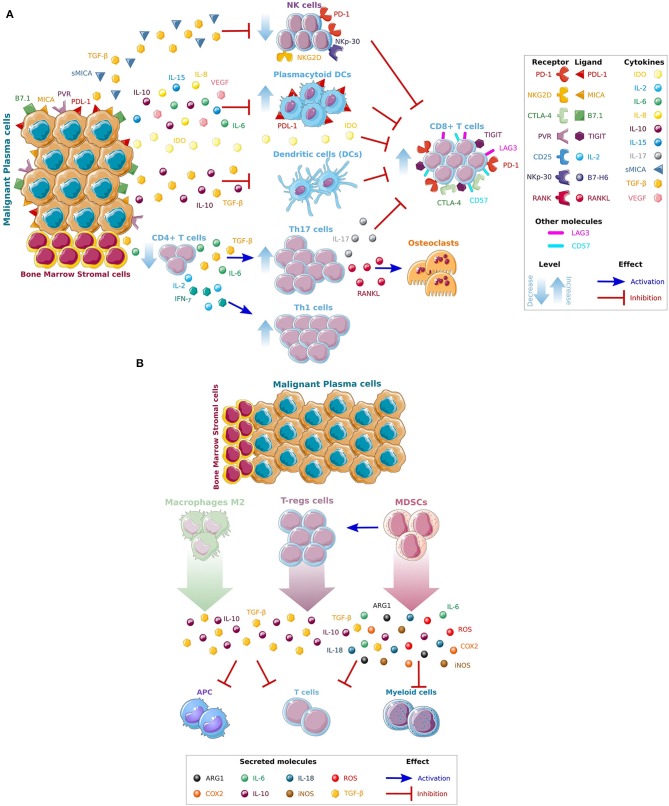
Mechanisms leading to MM immune escape. **(A)** Direct and indirect effects of MM cells on immune cells. The complex interactions of BM stromal cells and tumor cells, through the production of different cytokines and growth factors (IL6, IL8, IL10, IL15, VEGF) and immune inhibitors factors (sMICA,TGF-β, and IDO) released by MM cells, promotes MM growth and inhibit the activity of cytotoxic T (CD8+), dendritic (DC), and natural killer (NK) cells. **(B)** Recruitment of immunosuppressive cells in the BM microenvironment. An immunosuppressive microenvironment is also maintained by the recruitment of immunosuppressive cells such as macrophages M2, T-regs and Myeloid-derived suppressor cells (MDSCs) that further facilitate immune escape and promote disease progression. Interleukins IL-6, IL-8, IL-10, and IL-15, the vascular endothelial growth factor (VEGF), soluble major histocompatibility complex (MHC) class I chain-related protein A (sMICA), transforming growth factor beta (TGF-β), indoleamine 2,3-dioxygenase (IDO), T-helper (Th-), programmed death 1 (PD-1), cytotoxic T-lymphocyte-associated antigen 4 (CTLA-4), T-cell immunoglobulin and ITIM domains (TIGIT), lymphocyte-activation gene 3 (LAG-3), antigen presenting cells (APC), reactive oxygen species (ROS), cyclooxygenase-2 (COX2), and inducible nitric oxide synthase (iNOS). *Figures have been created using Smart Servier Medical Art website.

Along with the genomic changes occurring in plasma cells (11), the BM microenvironment supports MM progression, development of drug resistance and enable immune escape (6, 12, 13). It is composed of a cellular compartment (stromal cells, osteoblasts, osteoclasts, endothelial cells, and immune cells), the extracellular matrix components and soluble factors such as cytokines, chemokines, and growth factors. Immune cells are important component of the BM microenvironment. Several functional and numerical defects in the T-cells repertoire have been identified in MM patients. Reduced ratio of CD4:CD8 cells due to a reduction in the total number of CD4^+^ T-cells is one of the initial defects in parallel with an increase in the number of CD8^+^ T-cells (5). Of note, this ratio has been reported to decrease at the time of MM progression, and the reduction of CD4^+^ T-cells has been associated with progressed disease and poor prognosis (14). Significantly increased numbers of T helper (Th) type-1 (Th1), and type-17 (Th-17) have been also noticed in MM patients when compared to patients with monoclonal gammopathy of undetermined significance (MGUS) and healthy donors favoring a suppressive state (15, 16). Interleukin (IL)-6 and transforming growth factor-β (TGF-β) from the surrounding BM milieu play a critical role in the stimulation of Th-17. Th-17 are mainly enhanced in the BM milieu, where they are involved in MM bone disease due to the secretion of IL-17. By cooperating with the receptor activator of nuclear factor kappa-B ligand (RANKL) they can also activate osteoclasts and cause lytic lesions (17, 18). Normal in number but defective in their functions also the dendritic cells (DC) have been described to be altered in MM patients. After being stimulated by CD40 ligand, DCs are incapable of upregulating B7 co-stimulatory molecules such as CD80 and CD86 compromising the antigen presentation to the cytotoxic T-cells and repressing the recognition and killing of MM cells (19). TGF-β and IL-10 secreted by MM cells have been linked to this deficiency, that can be reverted back by adding IL-12 or interferon-γ (IFN–γ) (20). Of note, higher number of plasmacytoid dendritic cells (pDCs) have been reported in the BM of MM patients when compared with normal BM. They are knows to have high levels of programmed death 1 ligand (PD-L1) contributing to immune dysfunction and resulting in T-cell inhibition (21, 22). Furthermore, TGF-β secreted not only by MM cells, but also by regulatory T (Treg) cells and BM stroma cells lead to suppression of the number and function of circulating natural killer (NK) cells, which represent a key cellular subset of the innate immune system (23). The expression of the stimulatory NK receptors NKG2D and the natural cytotoxicity receptor NKp-30 are also reduced in MM patients causing a functional impairment of the NK cells (24). In addition, the secretion by the BM milieu of immune-modulating cytokines such as TGF-β and indoleamine 2,3-dioxygenase (IDO) have been also reported to create an immunosuppressive microenvironment. As such increased levels of these factors have been identified in the serum of MM patients (25, 26). Secretion of soluble molecules like soluble major histocompatibility complex (MHC) class I chain-related protein A (sMICA) by the malignant MM cells can also facilitate the inhibition of NK and CD8^+^ T-cells by downregulation of NKG2D and it has been linked to poor overall survival (27). Moreover, IDO is an enzyme that catalyzes the rate-limiting first step in the tryptophan catabolism causing tryptophan depletion in the BM milieu and subsequent inhibition of T cells function (28). A summary of the mechanisms leading to MM immune evasion in the BM microenvironment is shown in Figure 1A.

Lastly, an increase in the number of immunosuppressive cells such as Tregs, tumor-associated macrophages (TAMs) and myeloid-derived suppressor cells (MDSCs) have been demonstrated in advanced MM. Tregs express CD25 and transcriptional factor forkhead box P3 (FOXP3) and are capable of inducing expression of co-inhibitory molecules on antigen presenting cells (APCs). They are also known to secrete IL-10 and TGF-β and have the capacity to kill APCs and cytotoxic T-cells by using the granzyme- and perforin-dependent pathways (29). In MM TAMs are generally characterized as M2-like macrophages with limited cytotoxicity for tumor cells due to their reduced production of nitric oxide synthases (NOS) and pro-inflammatory cytokines. They have poor antigen-presenting capability and efficiently inhibit T-cell activation (30). Other suppressive cell type increased in MM patients are the MDSCs (31). They are a heterogeneous subset of immature myeloid progenitors cells able to inhibit both innate and adaptive immune responses and stimulate tumor growth (32). As such, they can inhibit T cell functions directly by generating arginases (ARG1), reactive oxygen species (ROS), cyclooxygenase-2 (COX2), inducible NOS (iNOS), and immunosuppressive cytokines (IL-6, IL-10, IL-18), as well as by reducing metabolic factors from the BM microenvironment required for T-cell activation (33). MDSCs have been also reported to inhibit effector T-cell responses by promoting T-reg cell development and by disrupting naive T-cell homing to lymph nodes (34). A summary of the recruitment of immunosuppressive cells in the BM milieu is shown in Figure 1B.

### T-Cell Exhaustion

Tumor-induced impairment of cytotoxic T-cells repertoire at the site of tumor has been also linked to immune escape and failure of immunotherapy-based approaches. An extensively investigated form of T-cell dysfunction is the T-cell exhaustion. Initially described in chronic viral infection and later in cancers, it results from prolonged antigen stimulation and is characterized by gradual loss of T-cells effector activity and increased expression levels of inhibitory receptors (35). To date, the importance of T-cell exhaustion in MM evolution has been ultimately assessed with limited agreement on whether CD8^+^ T-cells can be considered exhausted or senescent. Suen et al. (36) have analyzed the dysfunctional activity of clonal T-cells in MM and demonstrated that immunosenescence is the main feature of these cells. As such, they have showed that the T-cells presented a senescent secretory effector phenotype characterized by positive killer cell lectine like receptor (KLRG-1^+^)/CD57^+^/CD160^+^/CD28^−^ and low programmed death 1 (PD1) and cytotoxic T-lymphocyte-associated antigen 4 (CTLA-4) expression, proposing that in MM the T-cells have a molecular signature of senescence rather than exhaustion (36). In addition, Zelle-Rieser et al. (37) described that T-cells from the MM patients were severely more impaired in the BM than in the periphery (peripheral blood). They have demonstrated that at tumor site (BM), the CD8^+^ T-cells expressed several molecules associated with T-cell exhaustion such as PD1 and CTLA-4. In addition, these T-cells lack CD28 and are positive for CD57; a phenotype connected with inferior proliferative capacity and reduced function (37). Of note, Minnie et al. (38) have recently reported on the importance of immune suppression in enabling MM relapse after stem cell transplantation (SCT). They have demonstrated that BM-infiltrating CD8^+^ T-cells from MM-relapsed mice are phenotypically and functionally exhausted in RRMM patients following SCT. This was associated with increased production of IL-10 and increased expression of T-cell immunoglobulin and immunoreceptor tyrosine-based inhibition motif (ITIM) domains (TIGIT) and PD1 (38), highlighting the importance of early use of checkpoint inhibitions in MM to avoid T-cell exhaustion and provide a long-term immunological control.

## Strategies to Restore Immune Responses in Myeloma

### Agents That Remove the Breaks of the Immune System

#### Immunomodulatory Agents

Immunosuppression plays a critical role in MM pathogenesis, therefore reversing this suppression could potentially reinstate MM immune surveillance and improve disease control. IMiDs exhibit potent anti-MM activity (39–41). They possess pleiotropic properties including cytotoxic and immunomodulatory effects (42). In MM cells, through binding to cereblon (CRBN), an adaptor protein of the Cul4A-DDB1-ROC1 ubiquitin E3 ligase complex, they induce proteasomal degradation of the lymphoid transcription factors IKAROS family zinc finger 1 (IKZF1), AIOLOS (IKZF3) (43, 44) and casein kinase 1α (CK1α) (45) leading to the transcriptional repression of the interferon regulatory factor 4 (IRF4) and MYC. In an IKZF3 dependent mechanism, IMiDs stimulate T-cell proliferation and induce IL-2 and IFNγ secretion (44, 46). These cytokines increase the number of NK cells and improve their function to facilitate lysis of MM cells (47). IMiDs are also known to decrease the activity of T-regs (48) and enhance cytotoxic T lymphocyte (CTL, CD8^+^ T cells) activity against MM cells (49, 50). Furthermore, they improve tumor antigen uptake by DCs improving the efficacy of antigen presentation (51). These properties mark IMiDs as attractive backbone to use in combination with other anti-MM therapies, in particular the immune-based strategies. As such, in combination with monoclonal antibodies such as Elotuzumab targeting the glycoprotein SLAM family member 7 (SLAMF7), also known as CS1 (52, 53) or Daratumumab and Isatuximab targeting the CD38, IMiDs have showed significant synergistic effects, increase in overall response rate (ORR) and extension of progression-free survival (PFS) and overall survival (OS) in MM patients (54–56).

Of note, the synergistic phenomena reported when anti-CD38 antibodies are combined with IMiDs seem to derive from their co-modulated effects on the host adaptive and innate immunity, suggesting that the acquired resistance to this combination may be mainly immune-mediated. These CD38 targeting antibodies have been reported by many groups (including ours) to exert multiple anti-tumoral immune effect such as complement dependent cytotoxicity (CDC), antibody mediated cellular phagocytosis (ADCP) as well as antibody driven cellular cytotoxicity (ADCC) (57, 58). Previous work from our laboratory (59) has also demonstrated that in responding patients Daratumumab induces expansion of T-cells and increases T-cell clonality. In contrast, an increase of exhausted T-cells with upregulation of the checkpoint inhibitors lymphocyte-activation gene 3 (LAG3) and TIGIT was observed in resistant patients. Those findings identified T-cell exhaustion as cellular mediator of resistance to anti-CD38 antibodies and warrant further investigation of LAG3- and/or TIGIT-blocking approaches as possible ways to reinstate sensitivity to Daratumumab.

#### Immune Checkpoint Blockade

Co-stimulatory and co-inhibitory immune checkpoints tightly regulate the immune response upon activation to defend the host from autoimmunity or harm due to inflammation (60). Tumor cells are known to evade immune surveillance by upregulating ligands for inhibitory immune receptors triggering an exhaustion profile of T-cells (61). Efforts to reverse this exhausted phenotype have been achieved by blocking the inhibitors receptors expressed on T-cells though an immune checkpoint blockade. Instead of working directly on the tumor cells, these agents excite the host's immune system to induce an anti-tumor effect (62). Clinically, the most important checkpoints to date are CTLA-4 and PD-1/PDL-1 axis that have proven to be active in various solid tumors and hematologic malignancies (63, 64). In MM, although MM cells express PDL-1, the BM cytotoxic T-cells have low levels of PD-1, suggesting that PD-1 blockade may not be adequate to stimulate T-cells (65). Blocking antibodies targeting PD-1 (Pembrolizumab and Nivolumab) and PD-L1 (Durvalumab) have been assessed in MM. In monotherapy they did not show clinical responses, probably due to the described immune dysfunction reported in MM (66, 67). Better responses have been demonstrated in combination with IMiDs, due to the potential synergistic activation of adaptive and innate immunity (68, 69). However, those combinations were associated with various adverse events (AEs) (pulmonary, cardiac, gastrointestinal and hepatic toxicity) and an increased risk of death raising safety concerns. Therefore, while immune checkpoint inhibitors remain an attractive therapy for MM patients, further studies are required to increase clinical activity and limit the immune-mediated toxicity. The anti-PD-1 Cemiplimab is currently being evaluated in a phase I-II trial in combination with the anti-CD38 Isatuximab, whereas Durvalumab is under evaluation in combination with the other anti-CD38 Daratumumab. In addition, other checkpoint inhibitors (LAG-3, TIGIT, TIM-3 and CD85j) have been demonstrated relevant during MM progression (70–73) and antibodies against theses checkpoints inhibitors are in initial stages of development. A summary of combination trials with checkpoint inhibitors ongoing in MM is presented in Table 1.

**Table 1:** Summary of combination trials with checkpoint inhibitors ongoing in MM.

**Target**	**Type of therapy**	**Compound**	**Combination**	**Phase**	**NCT number**
PD-1	Immune checkpoint blockade	Cemiplimab	Isatuximab	I-II	NCT03194867
PD-1	Immune checkpoint blockade	Nivolumab	Pomalidomide and Dexamethasone	III	NCT02726581
PD-1	Immune checkpoint blockade	Nivolumab	Lenalidomide	II	NCT03333746
PD-1	Immune checkpoint blockade	Nivolumab	Daratumumab and Cyclophosphamide	II	NCT03184194
PD-1	Immune checkpoint blockade	Nivolumab	Dexamethasone, Carfilzomib, Nivoluman, and Reovirus	I	NCT03605719
PD-1	Immune checkpoint blockade	Pembrolizumab	Pomalidomide and Dexamethasone	I-II	NCT02289222
PD-1	Immune checkpoint blockade	Pembrolizumab	Lenalidomide and Dexamethasone	I	NCT02036502
PDL-1	Immune checkpoint blockade	Durvalumab	Daratumumab	I	NCT03000452

*The different combinations involving monoclonal antibodies against both PD-1 (pembrolizumab and nivolumab) and PD-L1 (durvalumab) are shown here. The clinical trial status and numbers are indicated here*.

Furthermore, since epigenetic abnormalities have been observed in cancer cells and tumor infiltrating T-cells (74) epigenetic modulating agents could be used to enhance anti-tumor immunity. DNA methyltransferase and inhibitors of histone deacetylase have been described to modify anti-tumor immune responses in numerous cancers (75, 76) including MM (77–79). Therefore, the potential synergy between epigenetic and immune therapies could also be further explored.

### Agents That Target Highly Selective Antigens on the MM Cells in the Form of Monoclonal Antibodies

Monoclonal antibodies (mAbs) have recently emerged as active therapeutic agents for the management of MM patients. They target highly selective antigens expressed in malignant plasma cells and not in normal tissues, stimulating specific anti-tumor activity and preventing toxicity due to off target effects. They elicit anti-MM activity through multiple mechanisms, including a direct cytotoxic effect on MM cells via apoptosis and an immune-mediated cytotoxicity such as ADCC, CDC, and ADCP. They can also be used to directly target the malignant plasma cells while releasing an anti-cancer agents linked via a chemical linker, as it is the case for the antibody-drug conjugates (ADCs), or to engage and stimulate cytotoxic T-cells for lysis of MM cells with bispecific T-cell engagers (BiTEs).

#### Anti-CS1/SLAMF7 Monoclonal Antibody

Elotuzumab is a humanized immunoglobulin (Ig) G_1_ monoclonal antibody that targets SLAMF7, also known as CS1, a glycoprotein greatly expressed in MM cells and NK cells (80). It mediates the killing of MM cells by NK cell-associated ADCC, NK cell activation and by inhibiting the interactions between MM cells and BM stromal cells (BMSCs) (81). Encouraging results were observed in RRMM patients treated with Elotuzumab when combined with IMiDs such as Lenalidomide and Pomalidomide (53, 82). The combination of Elotuzumab with Lenalidomide and Dexamethasone (Elo-Rd) were assessed in a phase III trial (ELOQUENT-2). In this trial, the triple regimen containing Elotuzumab demonstrated to be clinically superior than Lenalidomide and Dexamethasone (Rd) in terms of ORRs (79 vs. 66%), PFS (19.4 months vs. 14.9 months) and OS (48 vs. 40 months) without additional toxicity (53, 83). The separation of OS curves was also maintained over time in favor of Elo-Rd with 4 years OS rate of 50 vs. 43% for Rd (84). Elo-Rd is currently approved by both the Food and Drug Administration (FDA) and European Medicines Agency (EMA) for the treatment of RRMM patients after one line of prior therapy. A phase II trial of Elotuzumab in combination with Pomalidomide and Dexamethasone (Elo-Pd) vs. Pomalidomine and Dexamethasone (Pd) (ELOQUENT-3) in patients who received more than two previous therapies demonstrated that after a follow-up period of 9 months, Elo-Pd had a longer PFS (10.3 vs. 4.7 months) and a better ORR (53 vs. 26%) as compared to Pd alone (52). Furthermore, the Phase II trial of Elotuzumab plus Bortezomib and Dexamethasone (Elo-Bd) vs. Bortezomib and Dexamethasone (Bd) in patients who received from one to three prior therapies showed that Elo-Bd is also associated with longer median PFS (9.7 vs. 6.9 months). However, there was still no differences in ORR between these two groups (66 vs. 63%) (85, 86). Several studies are now ongoing to evaluate Elotuzumab-based combinations, such as Elotuzumab plus Lenalidomide, Bortezomib, and Dexamethasone (Elo-RVd), Elotuzumab plus Carfilzomib, Lenalidomide, and Dexamethasone (Elo-KRd) and Elotuzumab plus Pomalidomide, Bortezomib, and Dexamethasone (Elo-PVd). A summary of combination trials with anti-CS1/SLAMF7 ongoing in MM is shown in Table 2.

**Table 2:** Summary of combination trials with monoclonal antibodies ongoing in MM.

**Target**	**Type of therapy**	**Compound**	**Combination**	**Phase**	**NCT number**	**Trial name**
SLAMF7	Monoclonal antibody	Elotuzumab	Lenalidomideand Dexamethasone	III	NCT01239797	ELOQUENT-2
SLAMF7	Monoclonal antibody	Elotuzumab	Pomalidomide and Dexamethasone	II	NCT02654132	ELOQUENT-3
SLAMF7	Monoclonal antibody	Elotuzumab	Bortezomib and Dexamethasone	II	NCT01478048	
SLAMF7	Monoclonal antibody	Elotuzumab	Lenalidomide, Bortezomib, and Dexamethasone	II	NCT02375555	
SLAMF7	Monoclonal antibody	Elotuzumab	Kyprolis, Lenalidomide, and Dexamethasone	II	NCT02969837	
SLAMF7	Monoclonal antibody	Elotuzumab	Pomalidomide, Bortezomib, and Dexamethasone	II	NCT02718833	
CD38	Monoclonal antibody	Daratumumab	Lenalidomide and dexamethasone	III	NCT02076009	POLLUX
CD38	Monoclonal antibody	Daratumumab	Pomalidomide and Dexamethasone	II	NCT01998971	EQUULEUS
CD38	Monoclonal antibody	Daratumumab	Pomalidomide and Dexamethasone	III	NCT03180736	APOLLO
CD38	Monoclonal antibody	Daratumumab	Bortezomib and Dexamethasone	III	NCT02136134	CASTOR
CD38	Monoclonal antibody	Daratumumab	Carfilzomib and Dexamethasone	III	NCT03158688	CANDOR
CD38	Monoclonal antibody	Daratumumab	Bortezomib, Melphalan, and Prednisone	III	NCT02195479	ALCYONE
CD38	Monoclonal antibody	Daratumumab	Lenalidomide and Dexamethasone	III	NCT02252172	MAIA
CD38	Monoclonal antibody	Daratumumab	Bortezomib, Thalidomide, and Dexamethasone	III	NCT02541383	CASSIOPEIA
CD38	Monoclonal antibody	Daratumumab	Bortezomib, Lenalidomide, and Dexamethasone	II	NCT02874742	GRIFFIN
CD38	Monoclonal antibody	Isatuximab	Pomalidomide and Dexamethasone	III	NCT02990338	ICARIA
CD38	Monoclonal antibody	Isatuximab	Kyprolis and Dexamethasone	III	NCT03275285	IKEMA
CD38	Monoclonal antibody	MOR202		I	NCT01421186	
CD38	Monoclonal antibody	TAK-079		I	NCT03439280	

*The different combinations involving anti-SLAMF7 and anti-CD38 mAbs are shown here. The clinical trial status and numbers are indicated here*.

#### Anti-CD38 Monoclonal Antibodies

CD38 is a transmembrane glycoprotein highly expressed in MM cells and at low level in plasma, myeloid, and lymphoid cells, and some non-hematopoietic tissues (87). It has been reported to have ectoenzymatic activity and several functions in cell adhesion, signal transduction and calcium signaling (88, 89).

Daratumumab is the first anti-CD38 targeting antibody approved as monotherapy and in combination with numerous anti-MM standard regiments in MM. It is a fully humanized IgG_1_κappa monoclonal antibody that targets the cyclic ADP ribose hydrolase CD38. It mediates the killing of MM cells via CDC, ADCC, ADCP, and direct apoptosis via FcR-mediated cross-linking, and modulation of CD38 enzyme activities (57, 58, 90). Additionally, Daratumumab has showed an immunomodulatory role by promoting CD38^+^ immune regulatory cell and stimulating T-cell expansion. This process is associated with the increase of helper and cytotoxic T-cells, T-cell functional responses, and T-cell receptor (TCR) clonal expansion (91). Clinically, it demonstrated anti-MM activity both as monotherapy and when combined with novel agents in heavily pretreated RRMM patients. As such, the phase I GEN501 and phase II SIRIUS trials demonstrated that Daratumumab is effective as single agent in heavily pretreated patients and showed improved ORRs (29%), median PFS (mPFS) and OS of 3.7 and 17.5 months respectively (92–94). Based on these results FDA and EMA have now approved Daratumumab as single agent for RRMM patients who have received at least three prior lines of therapy including a proteasome inhibitors (PIs) and an IMiD.

In combination with Lenalidomide and Dexamethasone, Daratumumab (Dara-Rd) demonstrated significant efficacy in the phase III POLLUX trial. The ORR was 92.9% in Dara-Rd patients vs. 72.9 in Rd group, with a mPFS not reached vs. 17.5 months in the Dara-Rd vs. Rd arm and higher rate of patients achieving deep response with a minimal residual disease (MRD) negativity of 26% in the DRd group vs. 6% in the Rd group (54, 55, 95). Based on these data Dara-Rd is now approved for the treatment of MM patients who have previously received at least one line of therapy. In addition, Daratumumab has been used in combination with Pomalidomide and Dexamethasone (Dara-Pd). In the phase II trial EQUULEUS the three drug regimen showed an ORR of 60%, a mPFS and OS of 8.8 and 17.5 months respectively in heavily pretreated patients (96). Conclusive results will be derived from the ongoing phase III trial APOLLO, deigned to compare Dara-Pd vs. Pd in RRMM patients.

Daratumumab has been also combined with PIs. The phase III CASTOR trial revealed that adding Daratumumab to Bortezomib and Dexamethasone (Dara-Bd) resulted in higher ORR (83 vs. 63%), extended PFS (median 16.7 vs. 7.1 months) and higher MRD negativity rate (12 vs. 2%) (97–99). Based on these data the triplet Dara-Bd is also currently approved by the FDA and EMA for RRMM patients. In combination with Carfilzomib and Dexamethasone (Dara-Kd), a phase Ib showed that Daratumumab induced a clinical response in 84% of RRMM patients, previously receiving Lenalidomide and Bortezomib (100). Of note, a recent report on the ongoing phase III CANDOR study comparing Dara-Kd vs. Carfilzomib and Dexamethasone (Kd) demonstrated prolonged PFS with Dara-Kd vs. Kd (not reached vs. 15.8 months), ORR of 84.3 vs. 74.7% and higher MRD negativity rate (12.5 vs. 1.3%) (101). Based on these encouraging results Daratumumab is rapidly moving toward the first line treatment. As such, in older patients with newly diagnosed MM (NDMM) results from the phase III ALCYONE trial showed that Daratumumab in combination with Bortezomib, Melphalan and Prednisone (Dara-VMP) had impressive responses with an ORR of 91% vs. 74% with Bortezomib, Melphalan and Prednisone (VMP). At a median follow-up of 17 months an extended mPFS (not reached vs. 18.1 months) and increased MRD rate (22 vs. 6%) was reported with Dara-VMP as compared to VMP (99, 102). At median follow-up of 28 months, remarkable results in terms of higher MRD negativity (24.2 vs. 7.3%) and lower risk of progression or death (mPFS not reached vs. 32 months) were also observed in the phase III MAIA trial when Dara-Rd was compared to Rd in NDMM patients transplant-ineligible (95, 103). In NDMM patients eligible for Autologous SCT (ASCT), Daratumumab has also been used in combination with standard triplets such as Bortezomib plus Thalidomide and Dexamethasone (VTd), Bortezomib plus Lenalidomide and Dexamethasone (VRd), Dara-VTd and Dara-VRd. In the phase III CASSIOPEIA trial, following consolidation, the rate of MRD negativity was higher in the Dara-VTd group than in the VTd group (64 vs. 44%). These responses translated into a 53% reduction in the risk of progression or death for the Dara-VTd group vs. the VTd group (104) and led to the FDA approval of Dara-VTd as induction therapy for transplant eligible patients. VRd in combination with Daratumumab (Dara-VRd) has been also evaluated in the ongoing phase II trial GRIFFIN. Results presented at the last American Society of Hematology (ASH) 2019 meeting showed that the addition of Dara to VRd led to significant improvement in stringent complete remission (sCR) and depth of response when compared to VRd alone and did not affect the stem cell mobilization or hematopoietic reconstitution (105).

Isatuximab is a chimeric IgG_1−κ_ anti-CD38 mAb which selectively targets a unique epitope on human CD38 receptor and induces anti-MM activity by direct apoptosis, CDC, ADCC, and ADCP (106). Similarly to Daratumumab, Isatuximab demonstrated promising clinical activity in heavily pre-treated MM patients as single agent (107) and when used in combination with different anti-MM agents. In a phase Ib trial Isatuximab in combination with Rd showed an ORR of 51% and mPFS of 8.5 months in heavily pretreated patients of whom 68% had already received Carfilzomib or Pomalidomide (108). In combination with Pomalidomide and Dexamethasone the ORR was 62% and mPFs was 17.6 months (109). The phase III ICARIA trial comparing the triplet Isatuximab-Pomalidomide-Dexamethasone (Isa-Pd) to the duplet Pomalidomide-Dexamethasone (Pd) in RRMM patients receiving at least two previous lines of therapy is presently ongoing. Recently, a constant advantage in terms of ORR (60 vs. 35%) and mPFS (11.5 months vs. 6.5) was reported in the Isa-Pd group compared to the control group Pd at a median follow-up of 11.6 months, (56). The phase III IKEMA trial evaluating the combination of Isatuximab with Kd in RRMM patients is now ongoing and results awaited (110).

Of note, others anti-CD38 antibodies currently being evaluated include MOR202 (fully human from Morphosys), and TAK-079 (fully human from Takeda). Clinical activities in RRMM patients have been reported in combination with IMiDs (111, 112) and phase III trials are expected to start soon. A summary of combination trials with anti-CD38 mAbs ongoing in MM is presented in Table 2.

### Agents That Stimulate Immune Cells to Selectively Kill the Malignant Cells

#### Chimeric Antigen Receptor (CAR)—T Cell

In recent years, the development of therapeutic agents able to induce the autologous immune cells to mediate tumor cell killing and to overcome the immunosuppressive mechanisms of the tumor microenvironment has revolutionized the treatment of cancers. In this setting adaptive cell therapy using chimeric antigen receptor (CAR)-T cell therapy has been developed to eliminate cancer cells in many hematological malignancies including MM. CARs are artificial fusion proteins that consist of the extracellular antigen recognition part of an antibody from a single-chain variable fragment (scFv) fused with the CD3ξ chain for the intracellular signaling, and a T cell costimulatory domains (CD28 or 4-1BBB) (113). Via the scFv, CAR-transduced T-cells (CAR-T) can directly recognize and bind the antigen of interest, while the intracellular domain composed of the Cd3ζ chain of the T-cell receptor (TCR) induces T-cell activation (114). In contrast to a TCR, CAR-T cells are not restricted by MHC class and can recognize the antigen express on target cells independently of the MHC haplotype and the antigen presenting machinery.

Different antigen have been tested as targets for CAR-T cell therapy against MM. These include CD44v6, CD70, CD38, CD138, SLAMF7, and class C group 5 member D (GPRC5D) (115, 116). An important factor in the development of a successful CAR is selecting a proper surface antigen target that is absent in normal cells. To date the most promising antigen target is the B-cell maturation antigen (BCMA), and in this section we will focused on the different CAR-T cell therapies targeted this antigen in MM patients.

BCMA, a transmembrane signaling protein member of the tumor necrosis factor superfamily member 17 (TNFRSF17 or CD269), is expressed in mature B lymphocytes, and is important in maintaining the long-lived plasma cell homeostasis (117). It is also uniformly expressed in malignant plasma cells. Gamma-secretase directly shed BCMA from plasma cells (118), resulting in a soluble form that can be identified in peripheral blood where its serum levels correlate with response to therapy and overall survival (119). It binds to two cognate ligands B-Cell Activating Factor (BAFF) and A Proliferation Inducing Ligand (APRIL) leading to NF-kappaB and MAPK8/JNK activation and delivering critical survival signals for MM cells (120). As such, numerous anti-BCMA CAR-T cell therapies have been developed and showed impressive clinical activities in RRMM patients.

The first in-human trial using an anti-BCMA CAR-T cells was completed at the National Cancer Institute (NCI) by Brudno et al. (121). They have used a retrovirally transduced second generation of CAR generated by using a CD28 costimulatory domain and a murine scFv. At the highest dose the authors reported an ORR of 81%, with at least a complete response (CR) achieved in 13% of patients. Clinical responses and depth of response were positively correlated with the expansion of CAR^+^T-cell peak. Of note, cytokine release syndrome (CRS) of grade (G) 3–4 was not observed in the dose-expansion phase (121). Next, Cohen et al. (122) from the University of Pennsylvania in Philadelphia reported the clinical results of the phase I study conducted by using a fully human lentivirally transduced anti-BCMA CAR with the 4-1BB costimulatory domain. In the three cohorts enrolled on the study, the ORR was 44% in cohort 1, 20% in cohort 2, and 64% in cohort 3 and the mPFS was 2.2, 1.9, and 4.2 months respectively (122). Bb2121 is the second generation of anti-BCMA-CAR expressing the same scFc portion as the NCI trial with a 4-1BB costimulatory domain. Results from the multicenter phase I dose escalation trial testing bb2121 in RRMM have been recently reported and demonstrated an ORR of 85% with 45% of patients in CR or better. The clinical responses were quick with a median time to response of one month and all responding patients were MRD-negative. The median duration of response was 10.9 months and the mPFS was 11.8 months (123). The phase II single-arm trial evaluating the efficacy and safety of bb2121 in RRMM patients (KarMMa) has just finished enrollment and results awaited.

Following bb2121, the phase I trial of the next generation of anti-BCMA CAR-T therapy bb21217 has been also reported. The use of the phosphoinositide 3 kinase (PI3K) inhibitor bb007, to increase of memory-like CAR-T cells the final product, indicated similar ORR and toxicity profile to what was observed with bb2121. A longer follow-up and clinical data from patients receiving higher doses of cells are now required to understand if the *ex vivo* manipulation of the T cell products will result in enhanced efficacy (124).

To improve the effects of anti-BCMA CAR-T therapy, a CAR-T cell therapy targeting two different BCMA epitopes (VHH1 and VHH2) was recently developed (LCAR-B38M). It showed a high response rate, with an ORR of 88%, an MRD negativity of 63% and a median PFS of 15 months in RRMM patients (125, 126). Based on these clinical results, a phase Ib-II trial CARTITUDE-1 is currently ongoing in RRMM patients.

Additional CAR-T clinical trials targeting BCMA also include the JCARH125, MCARH171, and FCARH143 studies. They use three new CAR-T cell products composed of a human-derived scFv, a 4-1BB costimulatory domain, and a truncated human epidermal growth factor receptor (tEGFR), respectively, and are currently being evaluated in phase I clinical trials. A summary of combination trials with anti-BCMA CAR-T cell therapies ongoing in MM is presented in Table 3.

**Table 3:** Summary of combination trials with anti-BCMA T-cell therapies ongoing in MM.

**Target**	**Type of therapy**	**Compound**	**Combination**	**Phase**	**NCT number**
BCMA	CAR-T cell	Bb2121		I	NCT02658929
BCMA	CAR-T cell	Bb2121		II	NCT03361748
BCMA	CAR-T cell	Bb21217		I	NCT03274219
BCMA	CAR-T cell	LCAR-B38M		I	NCT03090659
BCMA	CAR-T cell	LCAR-B38M		Ib-II	NCT03548207
BCMA	CAR-T cell	JCARH125		I-II	NCT03430011
BCMA	CAR-T cell	MCARH171		I	NCT03070327
BCMA	CAR-T cell	FCARH143		I	NCT03338972
BCMA	ADCs	GSK2857916	Pembrolizumab	II	NCT03848845
BCMA	ADCs	GSK2857916	Pomalidomide	I-II	NCT03715478
BCMA	ADCs	GSK2857916	Lenalidomide/ Borthezomib and Dexamethasone	II	NCT03544281
BCMA	BiTE	AMG 420		I	NCT02514239
BCMA	BiTE	AMG 701		I	NCT03287908

*The different trials with anti-BCMA T-cell therapies (CAR-T and BiTEs) ongoing in MM are presented here. The clinical trial status and numbers are shown here*.

In addition to CAR-T therapy BCMA is also a perfect target for antibody-drug conjugates (ADCs) and for bispecific T-cell engagers (BiTEs). ADCs are immunoconjugates composed of a monoclonal antibody conjugated to a cytotoxic drug via a chemical linker. They precisely target cells expressing the target antigen and are then internalized to release the cytotoxic component and lead to cell death (127). Clinical results of the novel anti-BCMA-ADC conjugated to the antimitotic agent monomethyl auristatin F (GSK2857916) were recently reported. In heavily pre-treated MM patients, GSK285791 showed an ORR in 60% of patients with a mPFS of 12 months and 7.9 in patients refractory to both IMiDs and PIs (128). Additional trials are now evaluating its safety and efficacy in combination with Pembrolizumab, Pomalidomide, and Lenalidomide or Bortezomib in RRMM patients (Table 3).

#### Bispecific T-Cell Engagers

BiTEs are engineered molecules able to direct the host's immune system, more precisely the T-cells, against cancer cells. They are recombinant bispecific proteins with two linked scFvs. from two different antibodies, one targeting a cell-surface molecule on T cells (e.g., CD3ε) and the other targeting antigens on the surface of malignant cells. By binding to tumor antigens and T-cells simultaneously, BiTEs mediate T-cell responses and killing of tumor cells (129). Advantages of BiTEs include their ability to function independently of MHC haplotype and co-stimulation, and do not require peptide antigen presentation (130, 131). Furthermore, these molecules do not need *ex vivo* manipulation of T-cell and have relative simple production and purification allowing immediate treatment. The first approved BiTE was Blinatumomab, an anti-CD19, used for the treatment of relapse/refractory B cell acute lymphoblastic leukemia (ALL) (132).

In MM, among potential targets, BCMA, CD38, SMALF7, FcRH5, and G protein-coupled receptor (GPCR) GPRC5D, have been selected to develop anti-MM BiTEs, with BCMA representing the most promising target. As such, AMG 420 is the first anti-BCMA BiTE currently being evaluated in MM. It contains the scFv targeting BCMA in its N-terminal and CD3ξ in its C-terminal followed by a hexa-histidine (His6-tag) (133). Clinical results of the first-in-human dose escalation trial in RRMM patients, progressed after more than two lines of therapy, were recently presented. In this study AMG 420 induced an ORR of 70%, including 50% MRD-negative complete responses at the maximum tolerated dose (MTD) (134). A longer follow-up and more mature data are now needed to understand whether or not BiTEs will improve efficacy when compared to CAR-T therapy. Another anti-BCMA BiTE with an extended half-life and weekly short infusion, AMG 701, has showed significant activity in preclinical studies (135) and is currently being investigated in a phase I trial.

Research investigating tri-specific antibodies is also emerging. As such, HPN217 is the first in this category. It is designed to recognize human BCMA to target MM cells, serum albumin to extend its half-life, and CD3ε for the engagement of T cells. Preclinical studies have demonstrated BCMA- and T cell-dependent anti-tumor activity *in vitro* and in xenografts models of MM and lymphoma (136) and is currently under evaluation for further development and commercialization. A summary of combination trials with anti-BCMA BiTEs ongoing in MM is presented in Table 3.

#### Anti-MM Vaccination Approaches

Anti-cancer vaccines are based on the use of tumor antigens to stimulate the immune system and produce an antitumor response. To date, several therapeutic vaccine strategies have been established. These include the use of whole tumor cell, gene-modified tumor cells, or tumor-cell lysates, peptide or protein-based vaccines, RNA- and DNA- based vaccines, viral vector modified to express tumor antigen and DC-based vaccines containing DNA, RNA, or peptides (137, 138).

Numerous preclinical studies and clinical trials using these diverse therapeutic strategies have been completed and reported to be promising for the treatment of indolent metastatic disease (139, 140).

In MM these approaches have been used in disease stages with lower tumor burden including stem cell transplantation (SCT), precursor disease such as smoldering myeloma (SMM), and MRD settings (141). In the setting of transplantation studies have evaluated vaccines targeting hTERT, MAGE-A3, or survivin in combination with vaccine-primed autologous lymphocyte infusion (142–144). In SMM a multi-peptide vaccine PVX-140 has been designed to induce a T cell mediated immune response by specifically stimulating CTLs with the tumor antigen targets X-box binding protein 1 (XBP1), Syndecan-1 (CD138), and SLAMF7 (CS1). Memory CD8^+^ T-cell responses were reported and the vaccine demonstrated single-agent immunogenicity that was enhanced by the addition of Lenalidomide (145).

Among the cell-based vaccines, therapeutic strategies based on the use of autologous DCs pulsed with tumor antigens have been tested. As such, in a phase II trial, a fusion vaccine generated by combining autologous MM and DCs was administrated to MM patients following ASCT (146). It demonstrated a myeloma specific CD4 and CD8 T-cell responses with 24% of patients achieving a partial response (PR), that following vaccination, were converted to complete response (CR)/near CR (nCR), due to the effect of the vaccine on remaining disease (146). A clinical trial evaluating this fusion vaccine combined with a PD-1 inhibitor is now ongoing.

Overall, anti-MM vaccination therapy appears to be well tolerated and largely considered to have the greatest activity when used in combination with other therapies that have immunomodulatory properties. In this context, vaccines could increase the probability of clinical response or improve its duration making this approach a promising adjuvant strategy against MM.

## Discussion

The treatment of MM patients has improved significantly over the past few years (147). A better understanding of the immune-escape mechanisms contributing to tumor progression, has led to the development of several active and well-tolerated forms of immunotherapy that has significantly improved outcome of MM patients (9, 10).

IMiDs with their pleiotropic anti-MM properties have showed ability to enhance the effects of mAb treatments, checkpoint inhibitors and ADCs (55, 69, 84, 128). Other antibody-based immunotherapies, such as CAR-T and BiTEs, showed outstanding response rates in heavily pretreated patients (123, 134). However, relapses occur limiting the efficacy of this promising treatment approach. Lower expression of the targeted antigen on cell surface have been suggested as a possible mechanism of resistance to targeted mAb therapy (148, 149). Regarding CAR-T therapies, the composition and phenotypic of the administered T cells product, in term of the T-cells subsets, play a critical role for the clinical success of this strategy. Less differentiated (memory-like) CAR-T cells have been linked to better expansion, long-term *in vivo* persistence, and sustained anti-tumor control (124). Local immune suppression and T-cell exhaustion are important mediating factors of clinical outcomes and responses to immune-based approaches (59). Therefore, further characterization of the defects present in the immune system of MM patients is essential to develop novel therapies and to repurpose the existing ones.

Further research is now required to define the most active and safe combination and the most appropriate time point of drugs administration throughout the course of the disease. Although most of the immune-based studies were completed in RRMM patients, it is expected that patients benefit the most when it is used earlier in their disease course. The optimal sequence of the different type of immune therapies is also unspecified and in need of further studies.

Lastly, the detection of prognostic factors or biomarkers able to predict clinical responses and/or toxicity in patients will enable more active tailored treatments and better survival for MM patients. As such interrogation at single cell level of the BM immune repertoire of patients treated with immunotherapies can identify cellular mediators of sensitivity or resistance to those therapies and define potential means to reinstate sensitivity. Along with the improvement of existing therapeutic strategies and the development of new approaches, a better understanding of the role of immune system in MM pathogenesis is essential.

## Author Contributions

NL, RM, FH, and PN analyzed the data and wrote the manuscript.

## Conflict of Interest

The authors declare that the research was conducted in the absence of any commercial or financial relationships that could be construed as a potential conflict of interest.

## Abbreviations

ADCC: Antibody driven cellular cytotoxicity
ADCP: Antibody mediated cellular phagocytosis
ADCs: Antibody-drug conjugates
AEs: Adverse events
ALL: Acute lymphoblastic leukemia
APCs: Antigen presenting cells
APRIL: A proliferation inducing ligand
ARG1: Arginase
ASCT: Autologous stem cell transplant
ASH: American Society of Hematology
BAFF: B-Cell Activating Factor
BCMA: B-cell maturation antigen
BiTEs: Bispecific T-cell engagers
BM: Bone marrow
BMSCs: Bone marrow stromal cells
CAR: Chimeric antigen receptor
CDC: Complement dependent cytotoxicity
CK1α: Casein kinase 1α
COX2: Cyclooxygenase-2
CR: Complete response
CRBN: Cereblon
CRS: Cytokine release syndrome
CTL: Cytotoxic T lymphocyte
CTLA-4: Cytotoxic T-lymphocyte-associated antigen 4
DC: Dendritic cell
EMA: European Medicines Agency
FDA: Food and Drug Administration
FOXP3: Forkhead box P3
GPCR: G protein-coupled receptor
GPRC5D: Class C group 5 member D
His6-tag: Hexa-histidine
IDO: Indoleamine 2,3-dioxygenase
IFNγ: Interferon gamma
Ig: Immunoglobulin
IKZF1: IKAROS Family Zinc Finger 1
IKZF3: AIOLOS
IL: Interleukin
IMiDs: Immunomodulatory agents
iNOS: Inducible nitric oxygen synthase
IRF4: Interferon regulatory factor 4
ITIM: Immunoreceptor tyrosine based-inhibition motif
JNK: c-Jun N-terminal kinase
LAG3: Lymphocyte-activation gene 3
mAbs: Monoclonal antibodies
MAPK8: Mitogen-Activated Protein Kinase 8
MDSCs: Myeloid-derived suppressor cells
MGUS: Monoclonal Gammopathy of Undetermined Significance
MHC: Major histocompatibility complex
MM: Multiple myeloma
mOS: Median overall survival
mPFS: Median progression-free survival
MRD: Minimal Residual Disease
MTD: Maximum tolerated dose
NCI: National Cancer Institute
nCR: Near complete response
NK: Natural killer
NDMM: Newly diagnosed multiple myeloma
NOS: Nitric oxide synthase
ORRs: Overall response rates
OS: Overall survival
PD-L1: Programmed death-ligand 1
PD1: Programmed death 1
pDCs: Plasmacytoid dendritic cells
PFS: Progression-free survival
PI3K: Phosphoinositide 3 kinase
PIs: Proteasome inhibitors
PR: Partial response
RANKL: Receptor activator of nuclear factor kappa-B ligand
ROS: Reactive species of oxygen
RRMM: Relapsed refractory multiple myeloma
scFv: Single-chain variable fragment
sCR: Stringent complete remission
SCT: Stem cell transplantation
SLAMF7: Signaling lymphocytic activation molecule F7
SMM: smoldering multiple myeloma
sMICA: Soluble MHC class I chain-related protein A
TAMs: Tumor-associated macrophages
TCR: T-cell receptor
tEGFR: Truncated epidermal growth factor receptor
TGF- β: Transforming growth factor- β
Th: T helper
TIGIT: T-cell immunoglobulin and ITIM domains
TNFRSF17: Tumor necrosis factor superfamily member 17
Treg: Regulatory T cells
VEGF: Vascular endothelial growth factors
VH: Heavy chain variable domain
VL: Light chain variable domain
XBP1: X-box binding protein 1
Drug abbreviations: Elo-Rd: Elotuzumab with Lenalidomide and Dexamethasone
Rd: Lenalidomide and Dexamethasone
Elo-Pd: Elotuzumab plus Pomalidomide and Dexamethasone
Pd: Pomalidomine and Dexamethasone
Elo-Bd: Elotuzumab plus Bortezomib and Dexamethasone
Bd: Bortezomib and Dexamethasone
Elo-RVd: Elotuzumab plus Lenalidomide (Revlimid), Bortezomib (Velcade), and Dexamethasone
Elo-KRd: Elotuzumab plus Kyprolis (Carfilzomib), Lenalidomide, and Dexamethasone
Elo-PVd: Elotuzumab plus Pomalidomide, Bortezomib, and Dexamethasone
Dara-Rd: Daratumumab with Lenalidomide and Dexamethasone
Dara-Pd: Daratumumab with Pomalidomide and Dexamethasone
Dara-Bd: Daratumumab with Bortezomib and Dexamethasone
Dara-Kd: Daratumumab with Carfilzomib and Dexamethasone
Kd: Carfilzomib and Dexamethasone
Dara-VMP: Daratumumab with Bortezomib, Melphalan and Prednisone
VMP: Bortezomib, Melphalan and Prednisone
Dara-VTd: Daratumumab with Bortezomib plus Thalidomide and Dexamethasone
VTd: Bortezomib plus Thalidomide and Dexamethasone
Dara-VRd: Daratumumab with Bortezomib plus Lenalidomide and Dexamethasone
VRd: Bortezomib plus Lenalidomide and Dexamethasone
Isa-Pd: Isatuximab with Pomalidomide and Dexamethasone.
